# Sex difference in the association between blood alcohol concentration and serum ferritin

**DOI:** 10.3389/fpsyt.2023.1230406

**Published:** 2023-07-21

**Authors:** Asmaa Yehia, Ricardo A. L. Sousa, Osama A. Abulseoud

**Affiliations:** ^1^Department of Neuroscience, Graduate School of Biomedical Sciences, Mayo Clinic College of Medicine, Phoenix, AZ, United States; ^2^Department of Medical Physiology, Faculty of Medicine, Mansoura University, Mansoura, Egypt; ^3^Department of Psychiatry and Psychology, Mayo Clinic Arizona, Phoenix, AZ, United States

**Keywords:** alcohol intoxication, serum ferritin, liver enzymes, alanine aminotransferase, sex difference, blood alcohol concentration

## Abstract

**Introduction:**

The sex difference in alcohol use disorder (AUD) is ingrained in distinctive neurobiological responses between men and women, which necessitates further investigation for a more tailored management.

**Methods:**

Minding the findings of iron dysregulation in AUD and the sex difference in iron homeostasis in multiple physiological and pathological settings, we examined the sex difference in the association between serum ferritin and blood alcohol concentration (BAC) in intoxicated males (*n* = 125) and females (*n* = 59). We included patients with both serum ferritin tested of any value and a BAC above the level of detection during the same hospital admission period. We investigated sex difference in the relationship between BAC, serum ferritin and liver enzymes in intoxicated critically ill and noncritically ill patients.

**Results:**

We found a negative association between serum ferritin and BAC in critically ill, intoxicated females [*R*^2^ = 0.44, *F*(1,14) = 11.02, *p* = 0.005], with much attenuated serum ferritin in females compared to their male counterparts (194.5 ± 280.4 vs. 806.3 ± 3405.7 ng/L, *p* = 0.002). We found a positive association between serum ferritin and liver enzymes [alanine transaminase (ALT) and aspartate transferase (AST)] in critically ill intoxicated females [ALT: *R*^2^ = 0.48, *F*(1,10) = 9.1, *p* = 0.013; AST: *R*^2^ = 0.68, *F*(1,10) = 21.2, *p* = 0.001] and in noncritically ill intoxicated males [ALT: *R*^2^ = 0.1, *F*(1,83) = 9.4, *p* = 0.003; AST: *R*^2^ = 0.1, *F*(1,78) = 10.5, *p* = 0.002]. The effect of BAC on serum ferritin was not mediated by ALT [indirect effect: (*B* = 0.13, *p* = 0.1)]. We also found a significant effect of sex, anemia, intensive care unit (ICU) admission and mortality on serum ferritin.

**Discussion:**

Our results suggest that high BAC in intoxicated female patients is associated with attenuated serum ferritin levels, questioning the role of low serum ferritin in female vulnerability to alcohol.

## Introduction

Hazardous alcohol drinking is associated with numerous medical consequences and a substantial socioeconomic burden (1, 2). The prevalence of maladaptive drinking patterns and alcohol use disorder (AUD) has always been higher in men. However, over the past decade, this disparity in prevalence began to close in women’s favor, with nearly equal prevalence among adolescents (3–7). Sex difference in AUD is not only displayed in different prevalence but rather ingrained in distinctive neurobiological responses (8–10). Several studies show a telescoping effect in women since they exhibit more vulnerability to alcohol; with sooner progression to alcohol-related disorders at less drinking levels compared to men (11, 12). Nevertheless, this concept has been questioned by studies that showed shorter time to dependence in men, issuing the need for larger sample sizes and enrollment of more women (13).

Despite the controversy around sex difference in alcohol consumption-related clinical course, anatomical and pathological differences still hold true (9). In a 12 year prospective cohort study of almost 13,000 participants consuming alcohol, women, compared to men, displayed significantly higher risk of developing alcohol-induced liver injury and cirrhosis (14). In addition, in a study of 643 patients with alcohol dependence (225 females vs. 418 males), sub-regions of amygdala and hippocampus showed different volumes between both sexes (15). Several preclinical studies have shown female sex vulnerability to ethanol exposure with more neuronal injury, cell death and distinct alterations in neurotransmitters response and neuronal activities (16–18). As such, the underlying pathophysiology for sex difference in AUD and its related disorders is under intense investigation to achieve better understanding for the neurobiology and develop a more tailored and targeted treatment.

The intriguing relationship between alcohol consumption and iron homeostasis has received attention over the past decades (19). In a large study of 1,134 men and 2,241 women, both showed high levels of serum iron and serum ferritin specifically in subjects who had met criteria for alcohol dependence, suggesting that chronic alcohol intake has an effect on body iron stores (20). Similarly, Milman and Kirchgoff showed high serum ferritin concentration in healthy fit men (*n* = 1,044) and women (*n* = 1,191) with alcohol intake (21). Yet, the relationship between chronic alcohol drinking and iron homeostasis is complex. For example, while alcoholics are reported to have high serum ferritin and transferrin saturation (22), we still observe high prevalence of iron deficiency and iron deficiency anemia in these patients (23).

Several lines of evidence suggest that alcohol interferes with iron homeostasis. Dysregulated iron economy seems to contribute to a number of alcohol-related disorders, particularly those that affect the liver and the brain (24). Rich body of literature discusses the role of iron overload with alcohol consumption in alcohol-induced liver disease (ALD) (25–28). On the other hand, the effect of alcohol on brain iron has surprisingly received less attention. A small study in 20 males with AUD showed iron accumulation in basal ganglia and dentate nucleus (29). One recent large study utilized data from the United Kingdom Biobank to quantify brain iron content in a cohort of 20,729 patients showed higher brain iron deposition and worse cognitive functions in patients with moderate alcohol consumption (more than 56 g/week or roughly one drink/day) (30).

Subtle differences in iron metabolism between males and females have been documented under normal physiological conditions as well as during different neurological disorders such as normal aging-related cognitive decline, Alzheimer’s disease, multiple sclerosis, Parkinson’s disease, and stroke (31). Indeed, one study showed sex-specific association between brain iron content and cognitive decline in aging-related iron disturbance (32). In a study of healthy individuals (93 males and 72 females), there was a significant increase in brain ferritin iron in the basal ganglia and hippocampus associated with aging, however women showed much lower brain ferritin than men (33). Interestingly, the kidney and liver of aging female rats showed higher expression of ferritin than in males (34). Moreover, age-related changes of serum ferritin showed a sex difference in a study of 20,000 individuals (35).

Ferritin is a unique iron storage protein that has a fundamental role in iron homeostasis, including regulation of brain iron content. Ferritin has been a molecule of growing interest in the field of neurodegenerative disorders and neurotoxicity due to its pivotal role in the newly identified process of ferroptosis (36, 37). Ferroptosis is a form of iron-dependent cell death that has been recently pointed out in several animal studies as a potential culprit in alcohol-related complications, including acute ethanol exposure-induced cardiomyopathy (38), alcohol-induced liver injury (39), and alcohol-induced depression and anxiety (40). High serum ferritin was associated with ferritin degradation in hepatocytes in animal models exposed to alcohol, releasing ferrous iron, producing reactive oxygen species (ROS) through Fenton reaction, and activating ferroptosis (41, 42). The recent accounts on the relation between alcohol and ferroptosis and the involvement of ferritin requires taking a second look at alcohol-ferritin relationship.

This study aimed to shed light on the sex difference in the relationship between blood alcohol concentration and serum ferritin. The implication of clearing such a relation would be opening doors for studying the role of iron overload and ferroptosis in the sex difference in alcohol-related complications including cognitive impairment and neurotoxicity which would help directing AUD management towards a more targeted approach.

## Methods

### Patients and data collection

This study was approved by the Institutional Review Board (IRB) of the Mayo Clinic (ID#22-008591). All methods were performed in accordance with the relevant guidelines and regulations. Obtaining informed consent was waived by the IRB due to the retrospective nature of the study. Electronic medical records (EMR) of 184 adult patients (202 hospitalizations) who received medical care in the Mayo Clinic Health System (MCHS) from June 1, 2019, through June 1, 2022. We included patients in the study if they had both serum ferritin tested of any value and a blood alcohol concentration (BAC) above the level of detection (11 mg/dL) during the same hospital admission period. The following data were extracted from EMR: demographics, reason for hospitalization, alcohol use disorder, psychiatric and medical comorbidities, hospital course, BAC, serum ferritin, liver enzymes, intensive care unit (ICU) admission, and mortality.

### Statistical analysis

Continuous data were expressed as mean ± standard deviation while categorical data were expressed as percentage. The Kolmogorov–Smirnov and Shapiro–Wilk normality tests were used to test for normal distribution. To compare continuous variables between males and females, we used Student’s t test and Mann–Whitney U test for normally distributed and non-parametric data, respectively. The chi-square test was used to compare categorical variables. For pairwise comparison, Bonferroni correction was used for multiple comparisons. Linear regression was used to test the prediction effect of BAC and liver enzymes on serum ferritin after logarithmic transformation for the positively skewed ferritin, alanine transaminase (ALT), and aspartate transaminase (AST). We also ran a comparison between coefficients of correlation between serum ferritin and BAC in both males and females to further verify the sex difference, using Danielsoper calculator based on Fisher (43). Analysis of covariance (ANCOVA) was performed to test the effect of sex, ICU admission, mortality and anemia on serum ferritin using ALT as a covariate. Mediation analysis was used to test if ALT mediated the relationship between serum ferritin and BAC in the female group. Sobel test was used to test the significance of the indirect relationship between ferritin and alcohol mediated by ALT. Analyses were performed with SPSS V26 software (Armonk, NY: IBM Corp). Results were considered significant at *p* < 0.05.

## Results

### Patient demographics

A total of 184 patients with 202 hospitalizations were sorted into two groups based on their sex, with 67.9% (*n* = 125) males and 32.1% (*n* = 59) females. Both groups did not display significant differences in terms of their mean age (49.3 ± 14.2 in males vs. 49 ± 12.7 in females), race, ethnicity, employment and marital status, or history of current or past nicotine use (32.8% in males vs. 28.8% in females). The “female” group had a higher level of education since 6.8% of them had a Masters or PhD while none of the “males” group did (*p* = 0.003). Moreover, obesity [body mass index (BMI) of 30.1–40 kg/m^2^] was more predominant in males: 28.8% vs. 15.3% within females (*p* = 0.04; Table 1). Patients were hospitalized for alcohol-related reasons (intoxication, mild or severe use, withdrawal, or alcohol-related disorders; 36.5% in males vs. 41.5% in females) among other reasons (worsening psychiatric conditions, suicidal ideation, suicidal attempt, drug overdose, fractures, coronavirus disease 2019 (COVID-19) infection, sepsis, and others), with no significant difference between the two groups (Table 2).

**Table 1:** Demographics.

		Males (*n* = 125)	Females (*n* = 59)	Student t-test or Chi-square test (*p* value)
Sex [no (%)]		125 (67.9%)	59 (32.1%)	<0.001
Age (years) (Mean ± SD), range		49.3 ± 14.2 (24–83)	49 ± 12.7 (20–79)	*t* = 0.1, df = 182, *p* = 0.9
Race [no (%)]	White	99 (79.2%)	50 (84.7%)	0.4
	African American/African/Black	11 (8.8%)	2 (3.4%)	0.2
	American Indian/Alaskan Native	6 (4.8%)	2 (3.4%)	0.7
	Other	8 (6.4%)	1 (1.7%)	0.2
	Not provided	1 (0.8%)	4 (6.8%)	0.02
Non-Hispanic Ethnicity [no (%)]		112 (89.6%)	52 (88.1%)	0.8
Employment status [no (%)]	Unemployed	48 (38.4%)	28 (47.4%)	0.2
	Other (Employed/Student)	27 (21.6%)	14 (23.7%)	0.8
	Retired	21 (16.8%)	6 (10.2%)	0.2
	Disabled	10 (8%)	5 (8.5%)	0.9
	Self-employed	10 (8%)	1 (1.7%)	0.09
	Missing	9 (7.2%)	5 (8.5%)	0.8
Educational level (no (%))	≤12 grade or GED	13 (10.4%)	6 (10.2%)	0.9
	Bachelor’s or Professional school degree	13 (10.4%)	6 (10.2%)	0.9
	Some college	7 (5.6%)	7 (11.9%)	0.1
	Associate degree	7 (5.6%)	6 (10.2%)	0.3
	Masters or PhD	0 (0%)	4 (6.8%)	0.003
	Missing	85 (68%)	30 (50.8%)	0.03
Marital status [no (%)]	Single	67 (53.6%)	24 (40.7%)	0.1
	Married	30 (24%)	16 (27.1%)	0.6
	Divorced	16 (12.8%)	12(20.3%)	0.2
	Widow	3 (2.4%)	3 (5.1%)	0.3
	Separated	4 (3.2%)	2 (3.4%)	0.9
	Life-partner	2 (1.6%)	2 (3.4%)	0.4
	Missing	3 (2.4%)	0 (0%)	0.2
BMI (kg/m^2^) (Mean ± SD), range		28 ± 6.7 (14.1–49.7)	252 ± 5.8 (16.8.–44.6)	t = 2.6, df = 177, *p* = 0.009
BMI groups [no (%)]	<18 (kg/m^2^)	5 (4%)	4 (6.8%)	0.4
	18.1–30 (kg/m^2^)	77 (61.6%)	41 (69.5%)	0.3
	30.1–40 (kg/m^2^)	36 (28.8%)	9 (15.3%)	0.04
	>40 (kg/m^2^)	6 (4.8%)	1 (1.7%)	0.3
	Missing	1 (0.8%)	4 (6.8%)	0.02
History of current or past nicotine use [no (%)]		41 (32.8%)	17 (28.8%)	0.6
Patients with multiple hospitalization [no (%)]		10 (8%)	6 (10.2%)	0.6

**Table 2:** Reason for hospitalization.

		Males (*n* = 125) and 137 hospitalizations	Females (*n* = 59) and 65 hospitalizations	Chi-square test (*p* value)
Alcohol related	Any	50 (36.5%)	27 (41.5%)	0.5
	Intoxication	26 (19%)	13 (20%)	0.9
	Alcohol abuse/dependence	38 (27.7%)	18 (27.7%)	0.99
	Alcohol withdrawal	21 (15.3%)	9 (13.8%)	0.8
	Alcohol induced disorder	13 (9.5%)	4 (6.2%)	0.4
GIT related: bleeding/gastritis/pancreatitis		17 (12.4%)	10 (15.4%)	0.6
Psychiatric	Any	11 (8%)	7 (10.8%)	0.5
	Depression	2 (1.5%)	2 (3.1%)	0.4
	Anxiety	1 (0.7%)	0 (0%)	0.7
	Delirium	18 (14.4%)	5 (8.5%)	0.3
Suicide ideation or attempts including drug overdose		7 (5.1%)	5 (7.7%)	0.5
Cardiovascular related disorders		9 (6.6%)	0 (0%)	0.03
Fracture		3 (2.2%)	4 (6.2%)	0.2
SARS-CoV-2 positive test		5(3.6%)	1 (1.5%)	0.6
Sepsis		0 (0%)	2 (3.1%)	0.1
Diabetic complications		4 (2.9%)	1 (1.5%)	0.5
Hepatic disorders		3 (2.2%)	1 (1.5%)	0.6
Pulmonary disorders		3 (2.2%)	1 (1.5%)	0.6
Neurological disorders		4 (2.9%)	0 (0%)	0.3
Renal disorders		1 (0.7%)	1 (1.5%)	0.5
Others	All	21 (15.3%)	7 (10.8%)	0.4
	Weakness/Falling/Loss of consciousness	10 (7.3%)	1 (1.5%)	0.1
	Cellulitis/ Systemic inflammation/Contusions	4 (2.9%)	1 (1.5%)	0.5
	Dehydration/ Heatstroke	3 (2.2%)	1 (1.5%)	0.6
	Hypokalemia	1 (0.7%)	2 (3.1%)	0.2
	Anemia±Chronic blood loss	3 (2.2%)	0 (0%)	0.5
	Acidosis	0 (0%)	1 (1.5%)	0.3
	Anasarca	0 (0%)	1 (1.5%)	0.3

### Alcohol use disorder, psychiatric and medical comorbidities

Female patients exhibited a significantly higher prevalence of alcohol use, abuse or intoxication compared to males (27.1% vs. 11.2%, *p* = 0.006). However, there was no significant discrepancy as regards alcohol dependence, alcohol withdrawal syndrome, and alcohol-induced disorders. Psychiatric comorbidities were significantly predominant in 71.2% of females and 52% of males (*p* = 0.01). Although depression and anxiety showed a higher prevalence among females (47.5 and 44.1% vs. 30.4, 26.4% respectively, *p* = 0.02 for both), Schizophrenia, schizoaffective or bipolar disorder, delirium, and drug use were not significantly different between the two groups. Medical comorbidities were not significantly distinctive in the two groups. Nevertheless, a higher percentage of females showed anemia (62.7% vs. 34.4%, *p* < 0.001), specifically iron deficiency anemia (18.6% vs. 7.2%, *p* = 0.02; Table 3).

**Table 3:** Alcohol use disorder, psychiatric, and medical comorbidities.

			Males (*n* = 125)	Females (*n* = 59)	Chi-square test (*p* value)
Alcohol use disorder [no (%)]	Alcohol use or abuse with and without intoxication		14 (11.2%)	16 (27.1%)	0.006
	Alcohol dependence		81 (64.8%)	34 (57.6%)	0.3
	Alcohol withdrawal syndrome		35 (28%)	14 (23.7%)	0.5
	Alcohol-induced disorders		46 (36.8%)	21 (35.6%)	0.9
	Alcohol induced disorders	Hepatic (fatty liver, hepatitis, cirrhosis, fibrosis)	35 (28%)	18 (30.5%)	0.7
		Neurological (polyneuropathy, sleep disorder, delirium, degeneration)	9 (7.2%)	5 (8.5%)	0.8
		Pancreatitis (acute/chronic)	2 (1.6%)	2 (3.4%)	0.6
		Alcohol induced anxiety disorder/ mood disorder	2 (1.6%)	1 (1.7%)	0.96
		Gastritis	1 (0.8%)	1 (1.7%)	0.5
		Cardiomyopathy	4 (3.2%)	0 (0%)	0.3
		unspecified alcohol induced disorder	8 (6.4%)	2 (3.4%)	0.5
Comorbid psychiatric conditions [no (%)]	Comorbid psychiatric conditions		65 (52%)	42 (71.2%)	0.01
	Depression		38 (30.4%)	28 (47.5%)	0.02
	Anxiety		33 (26.4%)	26 (44.1%)	0.02
	Drug use	Any	19 (15.2%)	11 (18.6%)	0.5
		Cannabis	7 (5.6%)	3 (5.1%)	0.9
		Opioid	1 (0.8%)	2 (3.4%)	0.2
		Cocaine	1 (0.8%)	1 (1.7%)	0.5
		Poly-substance	5 (4%)	3 (5.1%)	0.7
		Unknown	6 (4.8%)	2 (3.4%)	0.6
	Delirium during this hospitalization		20 (16%)	8 (13.6%)	0.7
	History of schizophrenia or schizoaffective or bipolar disorder		9 (7.2%)	3 (5.1%)	0.7
Comorbid medical conditions [no (%)]	Anemia	Any	43 (34.4%)	37 (62.7%)	<0.001
		Iron deficiency anemia	9 (7.2%)	11 (18.6%)	0.02
	Cardiovascular	Any	65 (52%)	30 (50.8%)	0.9
		Hypertension	48 (38.4%)	16 (27.1%)	0.1
		Cardiomyopathy/Heart failure	13 (10.4%)	5 (8.5%)	0.7
	Neurological	Any	62 (49.6%)	28 (47.5%)	0.8
		Neuropathy	19 (15.2%)	10 (16.9%)	0.8
		Epilepsy/Seizures	11(8.8%)	3 (5.1%)	0.5
	GIT		58 (46.4%)	26 (44.1%)	0.8
	Hepatic		44 (35.2%)	24 (40.7%)	0.5
	Pulmonary		29 (23.2%)	20 (33.9%)	0.1
	Renal/Urinary		20 (16%)	15 (25.4%)	0.1
	Diabetes		21 (16.8%)	6 (10.2%)	0.2
	Thyroid disorders	Any	11 (8.8%)	7 (11.9%)	0.5
		Hypothyroidism	9 (7.2%)	4 (6.8%)	0.9
	COVID-19 positive during this hospitalization		12(9.6%)	3 (5.1%)	0.4
	Cancer		7 (5.6%)	5 (8.5%)	0.5

### Hospitalization, ICU admission, mortality, serum ferritin concentration, blood alcohol concentration, and liver enzymes

All patients were hospitalized with no significant difference in the hospital length of stay (males vs. females: 15.1 ± 22.5 vs. 9.4 ± 6.8 days). In addition, 27.7% of males and 24.6% of females required ICU admission with no significant difference. Both groups ended up with insignificantly different ICU length of stay, mortality rate (males vs. females: 15.2% vs. 23.7%) and hospital admission-to-death interval (males vs. females: 207.1 ± 206.8 vs. 250.9 ± 140.3 days). Both males and females shared high but non-significantly different values of BAC (males vs. females: 217.2 ± 131.7 vs. 221.8 ± 137.8 mg/dL). However, compared to females, males had significantly higher levels of serum ferritin (806.3 ± 3405.7 vs. 194.5 ± 280.4 ng/L, *p* = 0.002) and liver enzymes (ALT: 98.8 ± 132.1 vs. 36.1 ± 23.4, *p* < 0.001 and AST: 141 ± 190 vs. 86 ± 81.1, *p* = 0.07; Table 4).

**Table 4:** Hospital course, blood ethanol concentration, serum ferritin, liver enzymes, and mortality.

		Males (*n* = 125 and 137 hospitalizations)	Females (*n* = 59 and 65 hospitalizations)	Mann–Whitney test or Student t-test or chi square test (*p* value)
Hospital length of stay (days) (Mean ± SD) (range)		15.1 ± 22.5 (115)	9.4 ± 6.8 (21)	0.4
Required ICU admission [no (%)]		38 (27.7%)	16 (24.6%)	0.6
ICU length of stay (days) (Mean ± SD) (range)		5 ± 5.2 (26)	3.4 ± 2.1 (7)	0.3
blood alcohol concentration (mg/dL) (Mean ± SD)		217.2 ± 131.7 (13–536)	221.8 ± 137.8 (13–460)	*t* = 0.2, df = 200, *p* = 0.8
Ferritin concentration (ng/L) (Mean ± SD) (range)		806.3 ± 3405.7 (34283)	194.5 ± 280.4 (1384)	0.002
ALT Level (U/L) (Mean ± SD) (range)		98.8 ± 132.1 (669)	36.1 ± 23.4 (82)	<0.001
AST Level (U/L) (Mean ± SD) (range)		141 ± 190 (987)	86 ± 81.1 (291)	0.07
Alkaline Phosphatase (U/L) (Mean ± SD) (range)		129.7 ± 111.4 (796)	143.7 ± 115 (655)	0.2
Mortality (no (%))	All	19 (15.2%)	14 (23.7%)	0.2
	In hospital	1 (5.3%)	0 (0.00%)	0.4
	After discharge	18 (94.7%)	14 (100%)	0.4
Interval between hospital admission and death (days) (Mean ± SD)		207.1 ± 206.8 (2–765)	250.9 ± 140.3 (37–577)	*t* = 0.7, df = 33, *p* = 0.5

### The association between blood alcohol concentration and serum ferritin

**Figure 1:**
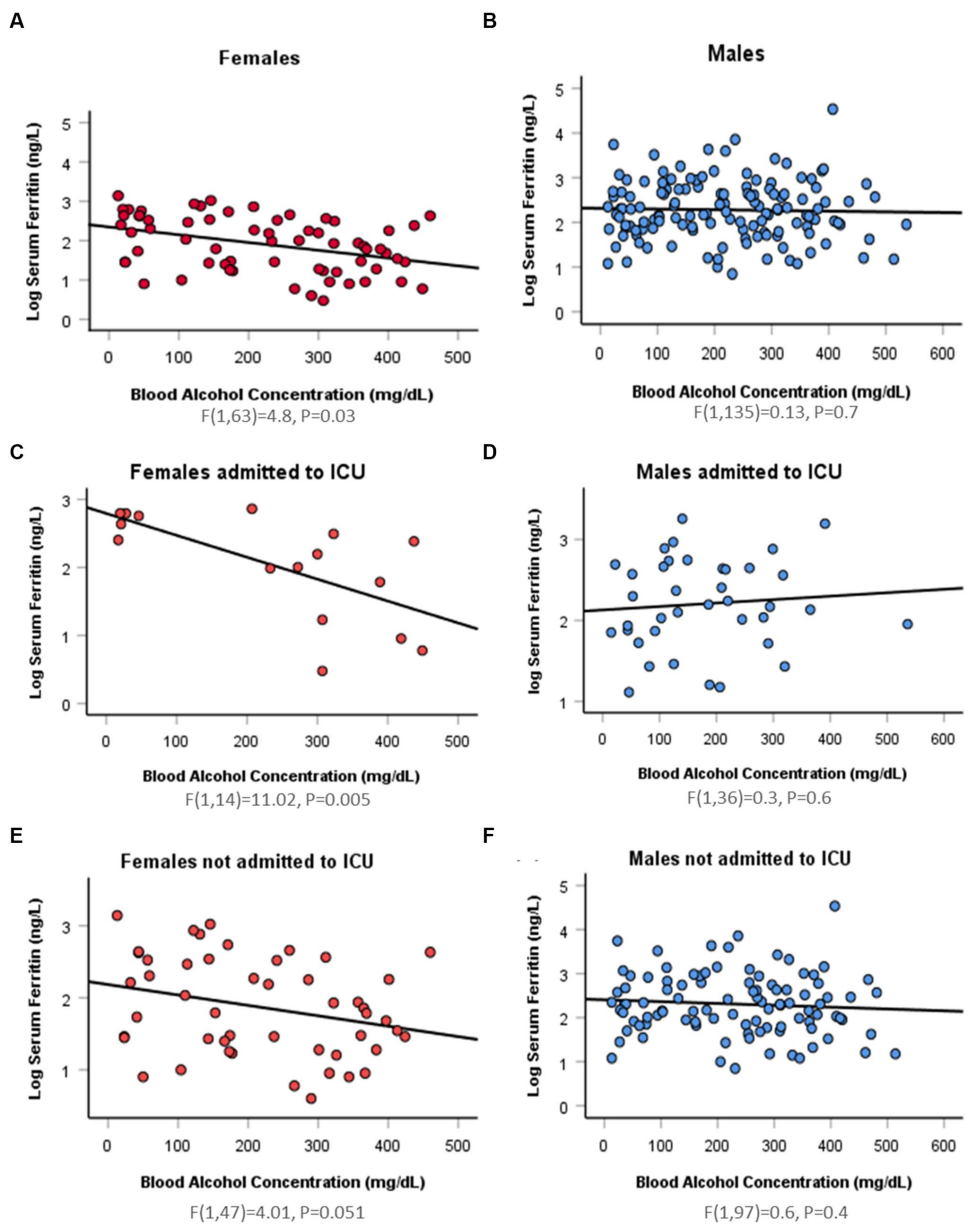
Sex difference in the association between log serum ferritin and blood alcohol concentration by linear regression. **(A)** A significant negative relation (*Beta =* −0.4) between log serum ferritin and blood alcohol concentration (BAC) detected in 65 hospitalizations for 59 intoxicated female patients [*F*(1,63) = 4.8, *p* = 0.03]. **(B)** An insignificant negative relation (*Beta =* −0.03) between log serum ferritin and BAC in 137 hospitalizations for 125 intoxicated male patients [*F*(1,135) = 0.13, *p* = 0.7]. **(C)** A significant negative relation (*Beta =* −0.7) between log serum ferritin and BAC in 16 ICU-admitted intoxicated female patients [*F*(1,14) = 11.02, *p* = 0.005]. **(D)** An insignificant positive relation (*Beta* = 0.09) between log serum ferritin and BAC in 38 ICU-admitted intoxicated male patients [*F*(1,36) = 0.3, *p* = 0.6]. **(E)** An insignificant negative relation (*Beta =* −0.3) between log serum ferritin and BAC in 49 hospitalizations for 43 non-ICU-admitted intoxicated female patients [*F*(1,47) = 4.01, *p* = 0.051]. **(F)** An insignificant negative relation (*Beta =* −0.08) between log serum ferritin and BAC in 99 hospitalizations for 87 non-ICU-admitted intoxicated male patients [*F*(1,97) = 0.6, *p* = 0.4].

Simple linear regression was used to assess if BAC significantly predicted serum ferritin concentration. In the female group, serum ferritin was negatively correlated with BAC (*r* = −0.4). Fifteen percentage of change in serum ferritin was accounted for by BAC (*R*^2^ = 0.15, *F*(1,63) = 11.2, *p* = 0.001). However, this finding varied when taking ICU admission into account. In ICU admitted females, 44% of change in serum ferritin was accounted for by BAC (*R*^2^ = 0.44, *F*(1,14) = 11.02, *p* = 0.005). While in non-ICU admitted females, BAC did not predict change in serum ferritin concentration (*R*^2^ = 0.1, *F*(1,47) = 4.01, *p* = 0.051). In the male group, there was no linear relationship between serum ferritin and serum ethanol whether patients were ICU admitted or not (Figure 1; Supplementary Table S1). On comparing correlation coefficients between males and females using Danielsoper calculator, we found a statistical difference with a z-Score = −2.6 and *p* = 0.01. On comparing coefficients in the ICU-admitted group, we also found statistical significance with a z-Score = −2.9 and *p* = 0.003. On the other hand, comparing coefficients in the non-ICU-admitted group showed no statistical significance, secluding the sex difference to the ICU-admitted group (z-Score = 0.28, *p* = 0.78).

### The association between liver enzymes and serum ferritin

In both groups, serum ferritin was positively correlated with serum ALT level (Females vs. Males: *r* = 0.3 vs. 0.3), and AST level (Females vs. Males: *r* = 0.4 vs. 0.3). In females, 12 and 17% of change in serum ferritin was accounted for by serum ALT (*R*^2^ = 0.12, *F*(1,43) = 5.9, *p* = 0.019) and serum AST (*R*^2^ = 0.17, *F*(1,40) = 8.2, *p* = 0.007) respectively. In males, 8% of variation in serum ferritin was accounted for by serum ALT (*R*^2^ = 0.08, *F*(1,115) = 10.2, *p* = 0.002) and 10% by AST (*R*^2^ = 0.1, *F*(1,106) = 11.4, *p* = 0.001). However, ICU admitted patients showed sex difference in serum ferritin variation predicted by liver enzymes. In the females group, 48 and 68% change in serum ferritin was accounted for by both ALT and AST, respectively, (ALT: *R*^2^ = 0.48, *F*(1,10) = 9.1, *p* = 0.013; AST: *R*^2^ = 0.68, *F*(1,10) = 21.2, *p* = 0.001). Nevertheless, no linear relationship was detected between serum ferritin and liver enzymes in ICU admitted males (Supplementary Table S2). Mediation analysis was used to test if the effect of BAC on serum ferritin was mediated through ALT in females. We did not perform mediation analysis in the male group due to absent significant association between BAC and serum ferritin. BAC was negatively correlated with serum ferritin (*B* = −0.4, *p* = 0.001). The effect of BAC on serum ferritin was not mediated by ALT [indirect effect: (*B* = 0.13, *p* = 0.1); Supplementary Table S3].

### The effects of sex, ICU admission, anemia, and mortality on serum ferritin

Using ALT as a covariate, ANCOVA showed a significant effect for sex: *F*(1,16) = 6.4, *p* = 0.01 on serum ferritin concentration with significantly higher ferritin in males (806.3 ± 3405.7 vs. 194.5 ± 280.4 ng/L, *p* = 0.002). In addition, there was a significant interaction between sex, ICU admission, and anemia [*F*(1,16) = 4.6, *p* = 0.03] and between sex, ICU admission, anemia, and mortality [*F*(1,16) = 7.6, *p* = 0.007; Supplementary Table S4].

## Discussion

The results of this study show high serum ferritin levels in both male and female alcoholics with much attenuated serum ferritin elevation in females. We also present an evident sex difference in the association between serum ferritin and BAC specifically in critically ill alcoholics. We detected a negative correlation between BAC and serum ferritin in critically ill intoxicated female patients, a relation that was not detected in non-critically ill ones. Interestingly, no such association between serum ferritin and BAC was detected in intoxicated male patients whether critically ill or not. Moreover, liver enzymes were much higher in male patients compared to their female counterparts despite no significant difference in BAC between the two groups. The association between serum ferritin and liver enzymes also revealed a sex difference and a distinction between critically ill and non-critically ill intoxicated males and females. It was crucial to look into this relation due to the remarkable role of the liver in hepcidin production, being the richest organ in iron content (44), and the first and major site for alcohol metabolism (45). Our study is the first to explore the sex difference in the relationship between BAC, serum ferritin and liver enzymes in intoxicated critically ill and non-critically ill patients. The demonstrated sex difference in ferritin response could come down to the sex difference in alcohol metabolism, alcohol-mediated oxidative stress, and alcohol-induced disruption of iron homeostasis, probably rendering females more vulnerable to ferritinophagy and ferroptosis; a possible mechanistic speculation that have prospects to explain the telescoping phenomenon in females and the sex difference in AUD neurobiological responses.

**Figure 2:**
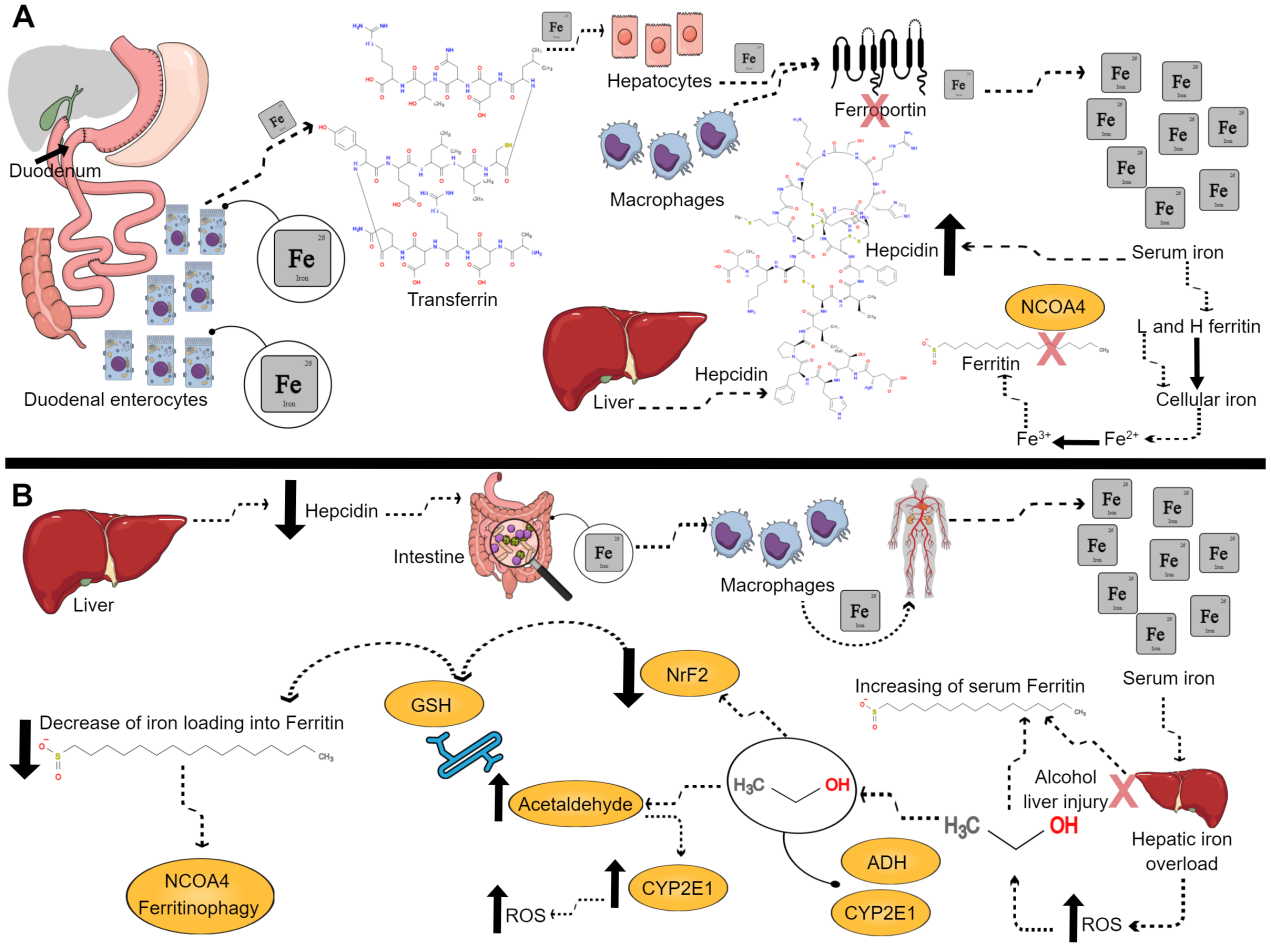
Ferritin under physiological and alcohol conditions. **(A)** Representative scheme of ferritin role under physiological conditions. Duodenal enterocytes absorb iron, which is carried on transferrin, and stored into hepatocytes, macrophages, and other cells. Iron exits these cells through ferroportin, which is degraded by liver produced hepcidin. Serum iron upregulates hepcidin. Cellular iron is loaded into apoferritin mediated made of H and L ferritin subunits. Iron loading is mediated by H ferritin which has a ferroxidase enzymatic activity to oxidize ferrous iron to ferric iron. When cells demand more iron, ferritin is degraded by nuclear receptor co-activator 4 (NCOA4) ferritinophagy, increasing available intracellular iron. **(B)** Representative scheme of ferritin role under alcohol intoxication. Alcohol down-regulates hepcidin, what leads to more intestinal iron absorption and release of iron from macrophages into the circulation favoring the increased levels of serum iron and leading to hepatic iron overload, which in turn leads to higher levels of ROS. Alcoholic liver injury upregulates ferritin synthesis, and, therefore, increases serum ferritin. Then, alcohol is metabolized by alcohol dehydrogenase (ADH) and cytochrome P450 2E1 (CYP2E1), oxidizing alcohol to acetaldehyde. The human CYP2E1 gene is up regulated by ethanol concentration. Thus, if there is more acetaldehyde formation that will lead to more CYP2E1 expression, and more reactive oxygen species (ROS) production. Acetaldehyde binds to glutathione (GSH), diminishing its antioxidant capacity. Alcohol decreases nuclear erythroid 2-related factor 2 (NrF2) and maximizes alcohol-induced oxidative stress by decreasing iron loading into ferritin. Low levels of NrF2 triggers NCOA4 ferritinophagy. In conclusion, there will be more free catalytic iron and more ferroptosis.

Due to the chaos that an excess of iron can create and the lack of a physiological mechanism for its excretion, iron is strictly regulated. Systemic iron pool builds up mainly from duodenal enterocytes absorbed iron, hepatocytes, and macrophages, with ferroportin as the only way out to exit these cells. Ferroportin is strictly controlled by hepcidin; a 25 amino acid peptide produced by the liver. Hepcidin expression, under physiological conditions, is up regulated by high serum iron. Hepcidin is considered the master hormonal regulator of iron since it controls the systemic availability of iron by limiting its intestinal absorption and blocking its cellular release through ferroportin degradation (46–48). Iron is transported in the circulation bound to liver-synthesized transferrin that allows iron entry into cells through transferrin receptors. In addition, iron is stored mainly in hepatocytes and macrophages of the reticuloendothelial system bound to ferritin (44). Apoferritin is an iron storage shell formed of 24 subunits of two types: Heavy (H ferritin) and Light (L ferritin). Iron loading into apoferritin is mediated by H ferritin that has a ferroxidase enzymatic activity to oxidize ferrous to ferric iron (47, 49). On the other hand, iron mobilization from ferritin is facilitated by ferritinophagy. Ferritinophagy is an autophagic degradation of ferritin mediated by nuclear receptor co-activator 4 (NCOA4), increasing iron cellular availability (50, 51). In cases of excess labile catalytic iron pool and over-activated ferritinophagy, H ferritin steps in to load iron into ferritin to prevent triggering ferroptosis (52, 53). Serum ferritin, secreted from hepatocytes, reticuloendothelial cells, and other parenchymal cells, has been always an indicator for body iron stores since it correlates with intracellular iron concentration in physiological settings and in cases of iron overload (Figure 2A) (54–56).

Our study displays high serum ferritin in both male (806.3 ± 3405.7 ng/L) and female intoxicated patients (194.5 ± 280.4 ng/L); a recurrent finding through the literature indicating the disruption of iron homeostasis associated with alcohol consumption and an increase in body iron stores (20, 21, 57–59). Alcohol down-regulates hepcidin which allows more intestinal iron absorption and release of iron from macrophages into the circulation, increasing serum iron which could be in part responsible for high serum ferritin levels, reflecting iron overload. Hepcidin down-regulation has been repeatedly documented as a trigger for hepatic iron overload, ROS production and liver injury in ALD (60–64). Nevertheless, several studies show unaltered or low serum iron with elevated serum ferritin in alcoholic patients, implying desensitization of hepcidin to iron level (19, 22, 23, 65, 66). In this case, high serum ferritin could be caused by *de novo* synthesis of ferritin through alcohol-induced inflammation (59, 67). While low serum iron could be due to alcohol-induced disruption and inflammation of intestinal mucosa which restricts iron absorption (68). Our study is limited by not providing serum iron or hepcidin measurements, however our results display anemia in 62.7% of females and 34.4% of males (*p* < 0.001) and iron deficiency anemia in 18.6% of females and 7.2% of males (*p* = 0.02).

Significantly attenuated high serum ferritin in intoxicated females compared to their male counterparts (806.3 ± 3405.7 vs. 194.5 ± 280.4 ng/L, *p* = 0.002) could possibly be explained by sex difference in hepcidin expression, ferritin synthesis or ferritin degradation. Animal studies have shown higher hepcidin expression in alcohol-fed females which could be caused by sex hormones or differences in ROS production (69). Hepcidin expression is controlled by hepcidin antimicrobial peptide (HAMP) gene in humans, and by Hepc1 and Hepc2 genes in mice. Harrison-Findik et al. found higher expression of Hepc1 in 20% ethanol fed female mice compared to males. Ethanol-mediated oxidative stress suppressed hepcidin, through inhibiting CCAAT/enhancer-binding protein α (C/EBPα), a transcription factor crucial for hepcidin expression, suggesting ROS mediated suppression of hepcidin in males (70). However, this notion could be contradicted by higher ROS production in female alcoholic patients and ethanol-fed animals (71–73). A more seemingly compelling argument for higher hepcidin in female alcoholics is the effect of sex hormones on hepcidin expression. Testosterone was found to down-regulate HAMP gene expression, while progesterone exerted an opposite effect, with contradictory reports on estrogen (74, 75). Nevertheless, higher hepcidin in females, compared to their male counterparts, could hamper iron intestinal absorption and iron cellular release, decreasing iron systemic availability, degrading ferroportin, and trapping ferritin inside the cells, which could account for lower serum ferritin levels.

In addition, lower serum ferritin levels in females could be simply explained by decreased ferritin synthesis in females due to lower iron stores caused by frequent blood loss (35) and the predominance of thyroid disorders in females (67, 76), with contradictory accounts on the effect of sex hormones on serum ferritin (77, 78). Moreover, ferritin degradation could account for the attenuated serum ferritin in females, a possibility that could be reinforced by the reported effect of sex difference on the process of ferroptosis (79) and the higher ROS production in female alcoholics (71–73). Further preclinical research is needed to investigate these different potential mechanisms behind sex difference in serum ferritin during alcohol intoxication.

Negative association between serum ferritin and BAC in intoxicated females could be the result of ferritin degradation with higher BAC, increasing labile catalytic iron which triggers ferroptosis and cell death, explaining in part female vulnerability to alcohol (11, 12). Female alcoholic patients display an exaggerated oxidative stress response to alcohol compared to males (73). This observation could be the result of sex difference in alcohol metabolism (80, 81). Oxidative ethanol metabolism in the liver, the primary site for alcohol metabolism, is implemented by alcohol dehydrogenase (ADH) and cytochrome P450 2E1 (CYP2E1), oxidizing alcohol to acetaldehyde. The human CYP2E1 gene is up-regulated by ethanol concentration, promoting acetaldehyde formation that triggers more CYP2E1 expression and ROS production (45, 82). Acetaldehyde production further compromises the antioxidant system through binding to glutathione (GSH), diminishing its antioxidant capacity (83). Nuclear erythroid 2-related factor 2 (NrF2), a transcription factor that up-regulates antioxidant gene expression, was decreased with chronic alcohol-feeding in rats, maximizing alcohol-induced oxidative stress (84). In addition, NrF2 was found to up-regulate H ferritin expression; a protective mechanism that helps iron loading into ferritin, preventing the detrimental cascade of ferroptosis triggered by free catalytic iron (42, 85–88). Moreover, the reduction of NrF2 was found to aggravate NCOA4 ferritinophagy (89). The process of alcohol metabolism seems to check all the boxes of ferroptosis characteristics; GSH depletion, ROS production, high lipid peroxidation products such as malondialdehyde (MDA) and 4-hydroxynonenal (4-HNE) and disruption of iron homeostasis (Figure 2B) (26, 37–40, 90).

An interesting animal study by Penaloza et al. (71) showed a sex difference in alcohol metabolism and alcohol-induced oxidative stress demonstrated by eight times higher mRNA expression of CYP2E1, a 15% higher ROS production and half GSH concentration in female mice compared to males (71). Based on these recent accounts, we propose that the exaggerated oxidative response in females to alcohol would overwhelm the antioxidant system, deplete GSH and down-regulate NrF2 which could decrease H ferritin production and promote NCOA4 ferritinophagy and ferroptosis, particularly with more ferritin trapped in the cells due to higher hepcidin expression. Therefore, lower serum ferritin in intoxicated females could indicate more vulnerability to alcohol-induced complications. Moreover, the distinction in serum ferritin-BAC association in our results between critically ill and non-critically ill intoxicated females (*p* = 0.051) implies the necessity to include a larger sample size. Nevertheless, this distinction could be explained by exaggerated state of oxidative stress in critically ill intoxicated female patients (91, 92). We also found a significant effect of the interaction between sex, ICU admission, anemia, and mortality on serum ferritin.

Our results show higher liver enzymes in male intoxicated patients which could be, in part, the result of a higher prevalence of elevated BMI (30.1–40 kg/m^2^; 28.8%) compared to female patients [15.3%, (*p* = 0.04)] (93). Values of liver enzymes could reflect the hepatocellular integrity. However, the prevalence of ALD or non-alcohol induced hepatic comorbidities was insignificantly different among intoxicated males and females in our study. Liver enzymes could be elevated as a response to excessive alcohol intake without evident liver insult in hazardous drinkers (94). This represents a limitation in our study since we did not characterize the amount of alcohol consumption. Nevertheless, our results showed that ALT did not mediate the effect of BAC on serum ferritin in females, indicating a much complex interaction between BAC and serum ferritin that extends beyond hepatic integrity. In addition, the sex difference in serum ferritin-liver enzymes association among critically and non-critically ill patients needs further characterization of age, blood pressure, waist circumference, plasma glucose, lipid profile, and white blood cells (WBC) count, since all can affect the association between serum ferritin and liver enzymes (95, 96).

Our findings support the current notion of closing the gap of AUD prevalence between men and women (3), with even higher prevalence of alcohol use with and without intoxication in women (27.1% vs. 11.2%, *p* = 0.006). Our data display a very high prevalence of psychiatric comorbidities which is a recurring theme among patients with AUD, probably due to shared psychopathological and neurobiological responses (97). In a recent work of ours, we detected attenuated serum ferritin levels in geriatric critically ill COVID-19 patients with psychiatric comorbidities compared to those with no psychiatric comorbidities, however no sex difference was found (98). This finding implies the need to consider the effect of psychiatric comorbidities on serum ferritin in intoxicated patients, especially since women displayed much higher prevalence of psychiatric conditions (71.2% vs. 52%, *p* = 0.01) in our current study. Moreover, we detected a high prevalence of long term post-hospitalization mortality in both sexes which is common among AUD patients, especially those with alcohol withdrawal syndrome, mostly due to high medical comorbidities and alcohol related disorders (99, 100) which is demonstrated in both males and females in our results.

One important factor that could affect serum ferritin among alcoholic patients is nicotine use, particularly with high prevalence of nicotine use among alcoholics (101). High serum ferritin has been reported in smokers (102, 103), interestingly showing a sex difference. One study found high serum ferritin levels in adolescent boys (*n* = 470) and girls (*n* = 379) with different types of smoking, compared to their non-smoker counterparts. In addition, they detected lower serum ferritin values among female smokers, compared to male smokers (104). The sex difference in serum ferritin reported in nicotine users could be explained in the light of the findings presented by Benowitz et al. They reported higher clearance and faster metabolism of nicotine in pre-menopausal women compared to men, particularly women on combined and estrogen-only contraceptives, implying a role for sex hormones in nicotine metabolism (105). Our study was limited by not considering the effect of nicotine on serum ferritin since our data on nicotine use was not clear whether it is for past or current use.

In conclusion, we found a negative association between serum ferritin and BAC in critically ill intoxicated females, with much attenuated elevation of serum ferritin in female alcoholics compared to their male counterparts. A possible underlying mechanism could boil down to sex difference in alcohol and iron metabolism. Exaggerated ROS production in females could overwhelm cellular antioxidant systems (e.g.; NrF2) which activates NCOA4 ferritinophagy and down-regulates H ferritin, causing ferritin degradation, decreasing iron loading into ferritin and releasing catalytic iron which promotes ferroptosis and cell death, particularly with trapped ferritin in the cells with higher hepcidin expression. The implications of verifying these mechanistic speculations would help signify ferroptosis and iron disruption as a culprit for sex difference in AUD and its related disorders, providing a more targeted approach for management and may be introducing low serum ferritin as a marker of alcohol vulnerability in intoxicated females.

### Limitations

The retrospective design of our study restricted the availability of data about peak serum ferritin, serum iron, serum hepcidin and other iron-related parameters such as transferrin saturation (TSAT) and total iron binding capacity (TIBC) which would have helped providing a clearer view of the disrupted iron metabolism with high BAC. We also could not provide data on oxidative stress and lipid peroxidation markers. We did not provide detailed description of the amount, duration and frequency of alcohol consumption which could be a confounding factor.

## Data availability statement

The raw data supporting the conclusions of this article will be made available by the authors, without undue reservation.

## Ethics statement

The studies involving human participants were reviewed and approved by Institutional Review Board of the Mayo Clinic (ID#22-008591). Written informed consent for participation was not required for this study in accordance with the national legislation and the institutional requirements.

## Author contributions

OA have full access to all the data in the study and takes responsibility for the integrity of the data and the accuracy of the data analysis, concept and design, and supervision. OA and AY acquisition, analysis, or interpretation of data. AY drafting of the manuscript. AY, RS, and OA critical revision of the manuscript for important intellectual content and administrative, technical, or material support. All authors contributed to the article and approved the submitted version.

## Funding

This work was funded by the department of Psychiatry and Psychology at the Mayo Clinic Arizona.

## Conflict of interest

The authors declare that the research was conducted in the absence of any commercial or financial relationships that could be construed as a potential conflict of interest.

## Publisher’s note

All claims expressed in this article are solely those of the authors and do not necessarily represent those of their affiliated organizations, or those of the publisher, the editors and the reviewers. Any product that may be evaluated in this article, or claim that may be made by its manufacturer, is not guaranteed or endorsed by the publisher.
