# Supplementary material for: Sex difference in the association between blood alcohol concentration and serum ferritin
Source: Front Psychiatry. 2023 Jul 21;14:1230406. doi: 10.3389/fpsyt.2023.1230406 (PMC10401063; doi:10.3389/fpsyt.2023.1230406)
Supplement: Supplementary file 1 [file Table_1.DOCX]

**Supplementary table S1: Sex difference in log serum ferritin in relation to blood alcohol concentration based on linear regression**

| Outcome (Log Serum ferritin) | Predictor | Beta | R square | df1 | df2 | F change | P value |
| --- | --- | --- | --- | --- | --- | --- | --- |
| Females | Blood alcohol concentration | -0.4 | 0.15 | 1 | 63 | 11.2 | 0.001* |
| ICU admitted females |  | - 0.7 | 0.44 | 1 | 14 | 11.02 | 0.005* |
| Non-ICU admitted females |  | - 0.3 | 0.1 | 1 | 47 | 4.01 | 0.051 |
| Males |  | - 0.03 | 0.001 | 1 | 135 | 0.13 | 0.7 |
| ICU admitted males |  | 0.09 | 0.008 | 1 | 36 | 0.3 | 0.6 |
| Non-ICU admitted males |  | - 0.08 | 0.006 | 1 | 97 | 0.6 | 0.4 |

**Supplementary table S2: Sex difference in log serum ferritin in relation to log liver enzymes based on linear regression**

| Outcome (Log Serum ferritin) | Predictor (liver enzymes) | Beta | R square | df1 | df2 | F change | P value |
| --- | --- | --- | --- | --- | --- | --- | --- |
| Females | Log ALT | 0.3 | 0.12 | 1 | 43 | 5.98 | 0.019* |
|  | Log AST | 0.4 | 0.17 | 1 | 40 | 8.2 | 0.007* |
| ICU admitted females | Log ALT | 0.7 | 0.48 | 1 | 10 | 9.1 | 0.013* |
|  | Log AST | 0.8 | 0.68 | 1 | 10 | 21.2 | 0.001* |
| Non-ICU admitted females | Log ALT | 0.2 | 0.037 | 1 | 31 | 1.2 | 0.3 |
|  | Log AST | 0.04 | 0.002 | 1 | 28 | 0.05 | 0.8 |
| Males | Log ALT | 0.3 | 0.08 | 1 | 115 | 10.2 | 0.002* |
|  | Log AST | 0.3 | 0.1 | 1 | 106 | 11.4 | 0.001* |
| ICU admitted males | Log ALT | 0.1 | 0.009 | 1 | 30 | 0.28 | 0.6 |
|  | Log AST | 0.15 | 0.02 | 1 | 26 | 0.57 | 0.5 |
| Non-ICU admitted males | Log ALT | 0.3 | 0.1 | 1 | 83 | 9.4 | 0.003* |
|  | Log AST | 0.3 | 0.1 | 1 | 78 | 10.5 | 0.002* |

**Supplementary table S3: Mediation analysis: ALT as a mediator between blood alcohol concentration and serum ferritin in females**

| Variable/ effect | b | SE | P |
| --- | --- | --- | --- |
| Ethanol → ferritin | - 0.002 | 0.001 | 0.02 |
| Ethanol → ALT | - 0.2 | 0.0003 | 0.1 |
| Ethanol → ALT→ ferritin | 0.65 | 0.34 | 0.06 |
| Effects | | | |
| Direct effect | -0.002 | 0.001 | 0.02* |
| Indirect effect | 0.13 | 0.07 | 0.1 |
| Total effect | -0.4 | 0.001 | 0.001* |

**Supplementary table S4: The effects of sex, ICU admission, mortality and anemia on serum ferritin based on ANCOVA**

| Variables | df | F change | P value |
| --- | --- | --- | --- |
| Sex | 1,16 | 6.4 | 0.01* |
| ICU admission | 1,16 | 1.7 | 0.2 |
| Anemia | 1,16 | 0.3 | 0.6 |
| Mortality | 1,16 | 0.1 | 0.8 |
| Sex*ICU admission | 1,16 | 0.3 | 0.6 |
| Sex*Mortality | 1,16 | 1.4 | 0.2 |
| Sex*Anemia | 1,16 | 2.5 | 0.1 |
| Anemia*Mortality | 1,16 | 0.03 | 0.8 |
| ICU admission *Mortality | 1,16 | 0.1 | 0.8 |
| ICU admission*Anemia | 1,16 | 0.05 | 0.8 |
| Sex*Anemia*mortality | 1,16 | 0.95 | 0.3 |
| Sex*ICU admission*mortality | 1,16 | 0.8 | 0.4 |
| Sex*ICU admission*Anemia | 1,16 | 4.6 | 0.03* |
| ICU admission*Anemia*mortality | 1,16 | 0.3 | 0.6 |
| Sex*ICU admission*Anemia*mortality | 1,16 | 7.6 | 0.007* |
| Log ALT (covariate) | 1,16 | 15.1 | <0.001* |
